# Single-cell RNA sequencing reveals dynamic changes in A-to-I RNA editome during early human embryogenesis

**DOI:** 10.1186/s12864-016-3115-2

**Published:** 2016-09-29

**Authors:** Si Qiu, Wenhui Li, Heng Xiong, Dongbing Liu, Yali Bai, Kui Wu, Xiuqing Zhang, Huanming Yang, Kun Ma, Yong Hou, Bo Li

**Affiliations:** 1 BGI Education Center, University of Chinese Academy of Sciences, Shenzhen, 518083 China; 2 BGI-Shenzhen, Shenzhen, 518103 China; 3 College of Life Science and Technology, Huazhong University of Science and Technology, Wuhan, 430074 China; 4 Department of Biology, University of Copenhagen, Copenhagen, 1599 Denmark; 5 James D. Watson Institute of Genome Sciences, Hangzhou, 310008 China; 6 BGI-Forensics, Shenzhen, 518083 China

**Keywords:** RNA-editing, Single cell transcriptome, Embryogenesis

## Abstract

**Background:**

A-to-I RNA-editing mediated by *ADAR* (adenosine deaminase acting on RNA) enzymes that converts adenosine to inosine in RNA sequence can generate mutations and alter gene regulation in metazoans. Previous studies have shown that A-to-I RNA-editing plays vital roles in mouse embryogenesis. However, the RNA-editing activities in early human embryonic development have not been investigated.

**Results:**

Here, we characterized genome-wide A-to-I RNA-editing activities during human early embryogenesis by profiling 68 single cells from 29 human embryos spanning from oocyte to morula stages. We demonstrate dynamic changes in genome-wide RNA-editing during early human embryogenesis in a stage-specific fashion. In parallel with *ADAR* expression level changes, the genome-wide A-to-I RNA-editing levels in cells remained relatively stable until 4-cell stage, but dramatically decreased at 8-cell stage, continually decreased at morula stage. We detected 37 non-synonymously RNA-edited genes, of which 5 were frequently found in cells of multiple embryonic stages. Moreover, we found that A-to-I editings in miRNA-targeted regions of a substantial number of genes preferably occurred in one or two sequential stages.

**Conclusions:**

Our single-cell analysis reveals dynamic changes in genome-wide RNA-editing during early human embryogenesis in a stage-specific fashion, and provides important insights into early human embryogenesis.

**Electronic supplementary material:**

The online version of this article (doi:10.1186/s12864-016-3115-2) contains supplementary material, which is available to authorized users.

## Background

A-to-I RNA-editing mediated by *ADAR* (adenosine deaminase acting on RNA) enzymes is the major RNA-editing that post-transcriptionally modifies nucleotide sequences on RNA molecules in metazoans [1]. RNA-editing can alter protein sequences, influence RNA stability and miRNA regulations in multiple biological processes including development and carcinogenesis [2]. The mammalian *ADAR* proteins include *ADAR*, *ADARB1*, and *ADARB2* [3].

Recent studies have demonstrated that most mice with a null allele of *ADAR* died before E14 due to defects in the hematopoietic system [4], and most mice with editing deficient *ADAR* mutation knock-in died at E13.5 as a result of unedited transcripts activating interferon and dsRNA sensing pathway [5]. Shtrichman et al. [6] found that editing levels of various target genes are substantially greater in most adult tissues than corresponding fetal ones and that *ADAR* protein is substantially regulated in undifferentiated pluripotent hESCs. These findings suggest that RNA-editing plays important roles in embryogenesis. Although early human embryonic transcriptome profiles have been studied [7–9], no research on RNA-editing activities before blastocyst stage during human embryogenesis has been conducted. To investigate RNA-editing activities during early embryogenesis in humans, we profiled the RNA editome from 68 single cells from 29 human embryos ranging from oocyte to morula stages using published human embryonic single cell transcriptome data [8, 9].

## Results

### Characteristics of RNA editome during early human embryogenesis

**Fig. 1:**
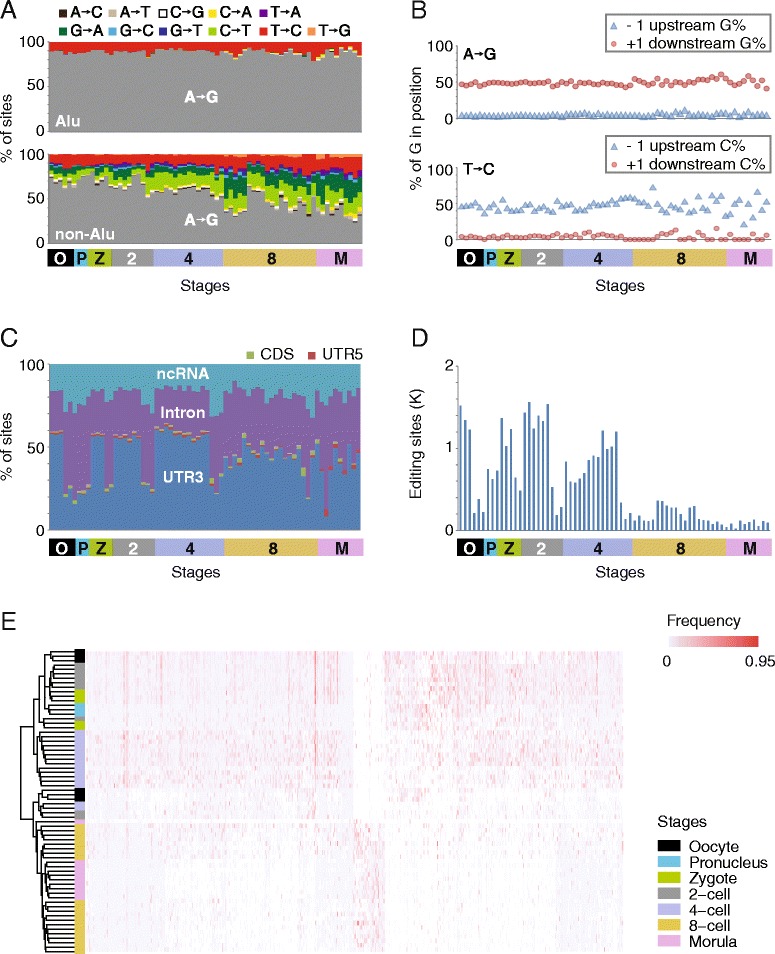
Characteristics of candidate RNA-editings in cells during early human embryogenesis. **a** Percentages of mismatch types in each cell. Each column represents one cell. **b** Neighbor preferences for A-to-G (top) and T-to-C (bottom) sites in each cell. A-to-G sites and the complementary strand of T-to-C sites display the motif signature of *ADAR* mediated A-to-I editing. Each pair of dots and triangles represents the G/C base % at position −1 (blue) and +1 (red) in one cell. **c** Percentages of A-to-I RNA-editing sites in functional genome elements at different stages. ncRNA, noncoding RNA. Each column represents one cell. **d** Changes in candidate A-to-I RNA-editing sites at different stages. Each bar represents one cell. O: oocyte; P: pronucleus; Z: zygote; 2: 2-cell; 4: 4-cell; 8: 8-cell; M: morula. **e** RNA-editing frequency at each editing site (column) in each cell (row). Blank regions, uniquely mapped reads < 4, not qualified for RNA-editing determination

By analyzing 68 single cells from 29 human embryos spanning from oocyte to morula stages in early embryogenesis (Additional file 1: Figure S1) using our RNA identification pipeline, we identified 14,049 candidate RNA mismatches, including 9,795 in Alu and 4,254 in non-Alu regions. Of the 9,795 mismatches in Alu regions we identified, A-to-G was the most prevalent mismatch type (account for 88.04 %), followed by T-to-C mismatches (account for 11.61 %), of which the majority were thought to be incorrect annotation of A-to-I editing because the RNA-seq libraries were not strand-specific [10]. The A-to-G and T-to-C mismatches together account for 99.65 % of the sites identified in Alu region (Fig. 1a). A typical *ADAR*-mediated editing is characterized by under-representation of G base in position −1 next to the edited site and over-representation of G base in position +1 next to the edited site [11]. Indeed, this characteristic was seen at our identified A-to-G sites and at the complementary strand of T-to-C sites (Fig. 1b). Of the 4,254 candidate RNA editing sites identified in non-Alu regions 2,247 were A-to-G mismatches (52.82 %) and 488 were T-to-C mismatches (11.47 %). The proportion of A-to-G/T-to-C mismatches in non-Alu regions is smaller than those in Alu regions (Fig. 1a). Similar findings have been reported by Fumagalli et al. [12] when they studied RNA-DNA single nucleotide differences (RDDs) in breast cancer. Considering that the proportion of validated RDDs was 90 % in Alu region and below 40 % outside Alu region in their study, and that the majority of editing events are in Alu elements in human [13], we only retain non-Alu A-to-G mismatches recorded in RADAR database [14] and Alu A-to-G mismatches as candidate A-to-I RNA-editing sites in our further analyses.

Totally, we identified 8,813 candidate A-to-I RNA-editing sites (Additional file 2: Table S1), of which 97.84 % located in Alu regions and 3,253 sites were present in the RADAR database. We annotated A-to-I RNA-editing sites and found that the majority were located in 3’-UTR regions (47.12 %), followed by intronic (33.77 %), non-coding RNA (ncRNA, 17.12 %), coding (0.98 %), and 5’-UTR regions (1.01 %), respectively (Fig. 1c). We noticed that the proportion of intronic A-to-I editing sites identified in our study is much smaller than that (94.03 %) in RADAR database, which may due to the low-coverage of the single cell RNA-seq data we analyzed. We will discuss this issue later on.

On average, we detected 819 ± 609, 702 ± 64, 953 ± 377, 1,057 ± 557, and 743 ± 330 A-to-I RNA-editing sites in each cell at oocyte, pronucleus, zygote, 2-cell, and 4-cell stages, respectively, whereas in cells at 8-cell and morula stages the A-to-I RNA-editing sites sharply dropped to only 190 ± 97 and 86 ± 35 per cell, respectively (Fig. 1d). To investigate the changes in RNA-editing patterns during human early embryogenesis, we clustered single cells based on editing frequencies (defined as the variant-supporting reads divided by all reads mapped to a specific RNA-editing site), and found that cells at 8-cell and morula stages were clustered together and separated from cells at earlier stages (Fig. 1e). The heatmap shows that ~15 % of A-to-I RNA-editing sites present in cells crossing from oocyte to 4-cell stages, but not in cells at 8-cell and morula stages, despite of expression of transcripts at moderate levels (Fig. 1e).

### A-to-I RNA-editing levels are sharply decreased in cells at 8-cell and morula stages

**Fig. 2:**
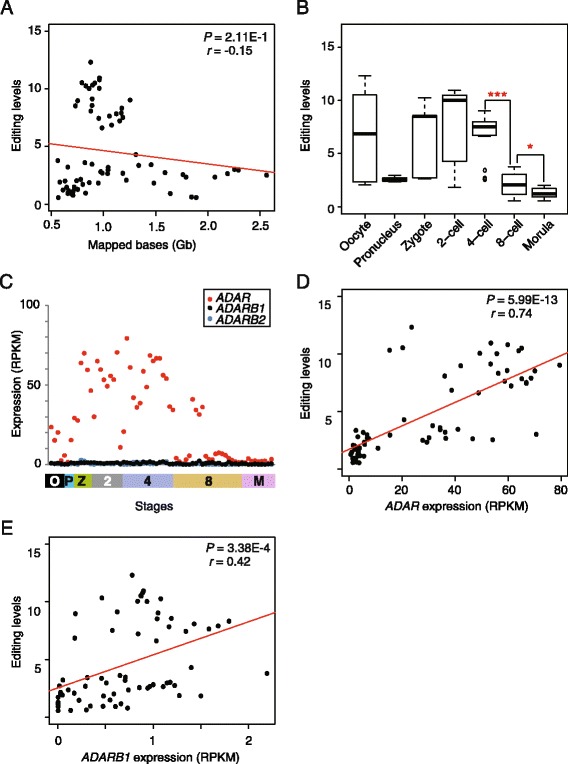
*ADAR*s govern changes in A-to-I RNA-editing levels during early human embryogenesis. **a** No correlation between editing level and mapped bases. **b** Changes in RNA-editing levels during early human embryogenesis. * *P* < 0.05; ** *P* < 0.01; *** *P* < 0.001 (Wilcoxon rank sum test). **c** Changes in gene expression of *ADAR, ADARB1*, and *ADARB2* during early human embryogenesis. Each colored dot (red, black, and blue) represents the gene expression levels of *ADAR, ADARB1*, and *ADARB2* in a cell. **d** and **e** Correlation between editing levels and the expression levels of *ADAR* and *ADARB1*. Each dot represents one cell

To profile genome-wide A-to-I RNA-editing changing patterns in the embryonic development, we defined A-to-I RNA-editing level as edited bases per million mapped bases in each cell. Under this definition, A-to-I RNA-editing levels are not affected by the number of mapped bases (Pearson’s correlation test, *P* = 2.11E-01, *r* = −0.15; Fig. 2a).

We observed that on average the RNA-editing levels remained relatively stable until 4-cell stage, but dramatically decreased (68 %) from 4-cell to 8-cell stages (Wilcoxon rank sum test, *P* = 7.92E-6), and continually decreased (41 %) at morula stage (Wilcoxon rank sum test, *P* = 1.48E-02; Fig. 2b and Additional file file 1: Figure S2). We then investigated the gene expression of *ADAR, ADARB1*, and *ADARB2* in cells at different stages, and found that *ADARB1* and *ADARB2* expression remained at low level (~1 Reads Per Kilobase per Million mapped reads, RPKM) across all embryonic stages investigated (Fig. 2c). The substantially lower expression of *ADARB1* than *ADAR* is also seen in many tissues in adult humans (Table S2) [15]. Amazingly, the changes in *ADAR* expression levels were almost in parallel with the changes in editing levels in cells at all stages investigated (Fig. 2b and Additional file 1: Figure S3). Correlation tests indicated that RNA-editing levels were strongly correlated with *ADAR* expression levels (Pearson’s correlation test, *P* = 5.99E-13, *r* = 0.74; Fig. 2d). Interestingly, we noticed the largest decreases in *ADAR* expression level and in A-to-I RNA-editing level occurred in the cells at 8-cell stage. It is worth noting that although the *ADARB1* expression levels remained low in cells of all stages investigated, we detected a moderate correlation between the *ADARB1* expression levels and the A-to-I RNA-editing levels (Pearson’s correlation test, *P* = 3.38E-4, *r* = 0.42; Fig. 2e).

### Non-synonymous A-to-I RNA-editing events frequently occurred in cells before 8-cell stage

**Fig. 3:**
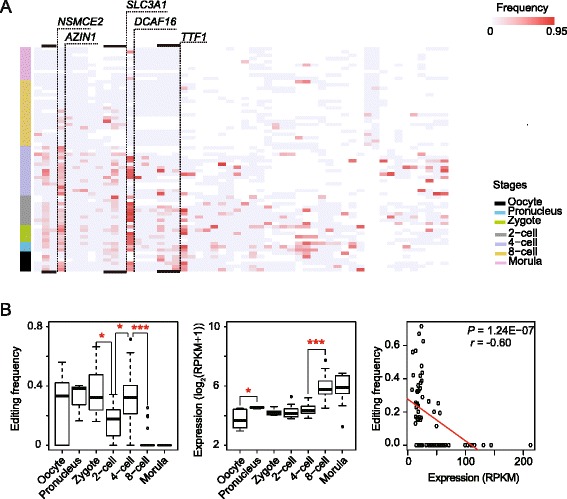
Recoding RNA-editing events in cells during early embryonic stages. **a** Heatmap of non-synonymous RNA-editing events in cells during early embryonic stages. Each row represents a cell, and each column represents a non-synonymous A-to-I editing site. Frequently edited genes are highlighted with dashed lines. Blank regions, uniquely mapped reads < 4, not qualified for RNA-editing determination. **b** Changes in non-synonymous editing frequency are negatively associated with the gene expression of *AZIN1.* Left, changes in *AZIN1*’s editing frequency; middle, changes in *AZIN1*’s expression; right, editing frequency is negatively associated with the *AZIN1* expression level, each open circle represents one single cell. * *P* < 0.05; ** *P* < 0.01; *** *P* < 0.001

We detected 324 A-to-I RNA editing events at 54 non-synonymous sites on 37 genes, of which 292 non-synonymous A-to-I RNA-editing events on 36 genes occurred in cells before 8-cell stage (Fig. 3a). Among the 54 nonsynonymous sites, 27 sites were deposited in RADAR (Additional file 2: Table S3). In addition, we observed that 7 genes were each non-synonymously edited at a specific embryonic stage.

We found that *AZIN1*, *DCAF16*, *SLC3A1*, *TTF1*, and *NSMCE2* were non-synonymously edited in more than 25 % of cells, almost exclusively confined to stages before 8-cell. Previous study showed that the non-synonymous RNA-editing sites in *AZIN1* (A1099G, ENST00000347770) and *DCAF16* (A486G, ENST00000382247) are clinically relevant in cancers [16]. We observed that the A1099G editing events on *AZIN1* occurred in 3 of 6 oocytes, 3 of 3 pronuclei, 5 of 5 zygotes, 6 of 9 2-cell cells, 14 of 15 4-cell cells, 4 of 20 8-cell cells and none in morulae cells, respectively, and the A486G editing events on *DCAF16* occurred in 3 of 6 oocytes, 3 of 3 pronuclei, 4 of 5 zygotes, 7 of 9 2-cell cells, 6 of 15 4-cell cells, none in 20 8-cell cells and 2 of 10 morulae cells, respectively. In 32 cells, we detected 41 non-synonymous A-to-I RNA-editing events at three sites (A1630G, A1646G, and A1691G, ENST00000409387) in the extracellular topological domain of *SLC3A1*, which encodes a renal amino acid transporter [17]. We found non-synonymous A-to-I RNA-editing events at three sites (A2651G, A2677G, and A2678G, ENST00000334270) altering the *TTF1* C-terminal amino acid sequence, which was suggested to mediate the termination of ribosomal gene transcription [18]. In addition, we found non-synonymous A-to-I RNA-editing events frequently altered the amino acid sequence of *NSMCE2* (A262G and A268G, ENST00000519712), which is required for DNA repair [19].

### Editing frequencies at the exonic RNA editing sites of four genes are negatively associated with their expression

We conducted Spearman’s correlation analysis between gene expression and editing frequency on 163 genes which were edited in the exon regions and found that A-to-I RNA-editing frequencies were negatively associated with the expression of four frequently edited genes (adjusted *P* < 0.1, Benjamini-Hochberg method). For instance, the frequency of non-synonymous editing A1099G on *AZIN1* dramatically dropped from 4-cell to 8-cell stages (Wilcox rank sum test, *P* = 2.67E-6; Fig. 3b) while the *AZIN1* expression substantially increased (edgeR, *P* = 3.24E-17; Fig. 3b). We found that *AZIN1* expression was highly negatively associated with editing frequency of A1099G (Spearman’s correlation test, *r* = −0.60, adjusted *P =* 2.02E-05, Fig. 3b). This pattern was also seen in protein coding genes *DCAF16* (Spearman’s correlation test, *r* = −0.43, adjusted *P =* 1.99E-02, Additional file 1: Figure S4A), and in long intergenic noncoding RNAs *RPL23AP53* (Spearman’s correlation test, *r* = −0.58, adjusted *P =* 2.63E-03, Additional file 1: Figure S4B) and *SNHG16* (Small Nucleolar RNA Host Gene 16) which was suggested to be associated with cell proliferation [20] (Spearman’s correlation test, *r* = −0.57, adjusted *P =* 4.66E-05, Additional file 1: Figure S4C).

### A-to-I RNA-editing events in miRNA-targeted mRNA regions of many genes preferably occur at specific stages

It has been suggested that RNA-editing in miRNA-targeted regions could alter miRNA-mediated post-transcriptional gene silencing [21] and gene expression [22]. We identified 609 A-to-I RNA-editing sites in miRNA-targeted regions on 298 genes (Additional file 2: Table S4), and noticed that the editing sites are relatively stable in cells from oocyte to 4-cell stages (57 ± 51, 25 ± 5, 62 ± 39, 68 ± 43, 61 ± 29 in oocyte, pronucleus, zygote, 2-cell, and 4-cell stages, respectively). However, the editing sites in cells at 8-cell and morula stages sharply dropped to 13 ± 8 and 4 ± 2, respectively (Additional file 1: Figure S5). We noticed that the editing events in miRNA-targeted regions on 114 genes, enriched in cell cycle (GSEA, adjusted *P* = 5.94E-08, Additional file 2: Table S5), occurred preferably in cells crossing oocyte to 4-cell stages (Fisher’s exact test, *P* < 0.05).

**Fig. 4:**
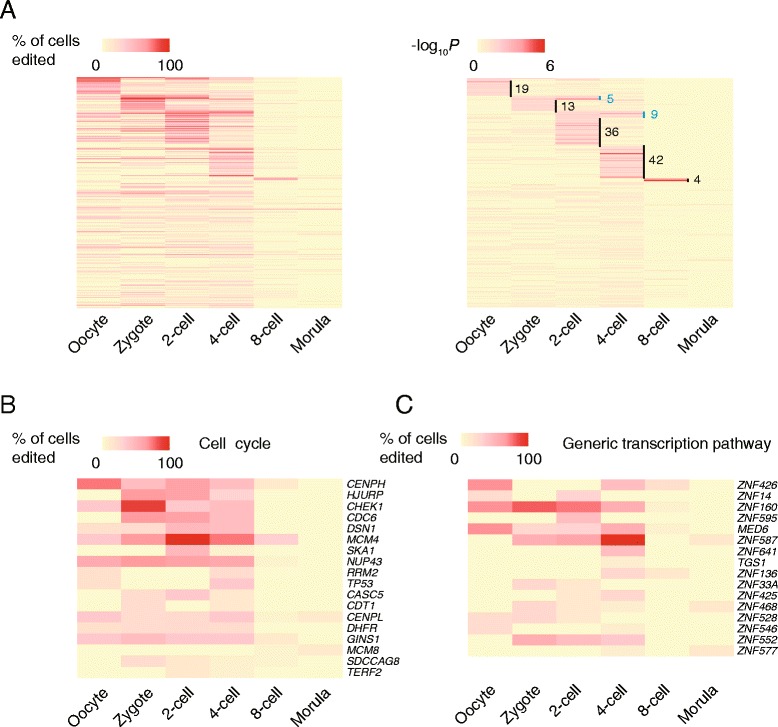
Genes are edited in miRNA-targeted mRNA regions in a stage-specific fashion. **a** Heatmaps of the percentage of cells edited at each stage (left) and *P*-values by Fisher’s exact tests (excluding pronucleus stage for insufficient cells) on the number of edited and unedited cells between a specific stage and other stages (right). Vertical bars highlight the genes that are more frequently edited in miRNA-targeted regions at one (black) or two sequential stages (blue) than other stages. Arabic numeral denotes the number of genes in each group. **b** and **c** Heatmaps of the frequencies of cells edited on genes involved in cell cycle and in generic transcription pathway, respectively. Each column represents an embryonic stage, and each row represents a gene

In addition to the differences in quantities of editing sites at different stages, A-to-I RNA-editing in miRNA-targeted regions on a substantial number of genes appears to be stage related. By calculating the percentage of cells with editing events in miRNA-targeted regions on each gene at each stage, we found that a group of 19 genes were more frequently edited in oocytes than in cells of other stages (Fisher’s exact test, *P* < 0.05), whereas a different group of 13 genes were more frequently edited in zygotes (Fisher’s exact test, *P* < 0.05), and another group of 36 genes were more frequently edited in cells at 2-cell stage (Fisher’s exact test, *P* < 0.05). Interestingly, the number of genes that were more frequently edited in miRNA-targeted regions increased to 42 in cells at 4-cell stage but suddenly decreased to 4 in cells at 8-cell stage (Fig. 4a). In addition, we observed a small number of genes that were more frequently edited in miRNA-targeted regions in cells crossing two sequential stages than other stages. For instance, one group of 5 genes were more frequently edited at zygote and 2-cell stages, while another 9 genes were more frequently edited at 2-cell and 4-cell stages (Fig. 4a). We noticed that 16 genes involved in generic transcription pathway and 18 genes involved in cell cycle, were edited in miRNA-targeted regions at most stages (Fig. 4b and c). Interestingly, *NUP43* and *MCM4* involved in cell cycle were frequently edited in miRNA-targeted regions in cells across zygote to 4-cell stages (Fig. 4b).

## Discussion

In this study, we detected genome-wide A-to-I RNA-editing in cells ranging from oocyte to morula stages, and defined editing-level to reflect RNA-editing activities in a cell. We showed that A-to-I RNA-editing levels dramatically decreased at 8-cell stage. By looking at the impacts of A-to-I RNA-editing on protein recoding, we found that seven genes such as *AZIN1* were frequently non-synonymously edited in cells of multiple embryonic stages.

We noticed that the proportion of intronic A-to-I editing sites identified in our study (33.70 %) is much smaller than that (94.03 %) in RADAR. Because introns are retained only in premature mRNA of genes, the sequencing depths in intron regions are usually lower than those in exon region of expressed genes in RNA-seq. Because the single cell RNA-seq data we analyzed are low-coverage, thus, the depths of many intronic regions are less than 4, which do not meet our requirement for determination of RNA editing events. This leads to a substantial reduction in A-to-I RNA editing sites being identified in intron regions, consequently largely reducing the percentage of intronic A-to-I RNA editing sites among the total A-to-I RNA editing sites identified.

Human embryogenesis is a complex and genetically well-programmed developmental process that is controlled by cascades of genes. Our results indicated that A-to-I RNA-editing acted in a stage-specific fashion during human early embryogenesis. We noticed that genome-wide A-to-I editing level suddenly and dramatically dropped in cells at 8-cell stage, suggesting that this sudden drop of A-to-I RNA-editing level may have an important biological significance during human early embryogenesis. Although the biological function of this sudden drop of editing level is yet to be discovered, we consider this event particularly interesting. Previous studies suggested that the 8-cell stage is a turning point because many important biological events occur at this stage. For example, a recent study on human early embryogenesis showed that there was a dramatic change in gene expression in cells at 8-cell stage as compared to the previous stages. At 8-cell stage, the expression of 3037 genes, enriched with genes involved in regulation of transcription and regulation of RNA metabolic process, substantially up-regulated while 1941 genes, enriched with genes involved in regulation of transcription and cell cycle, substantially down-regulated [9]. The X chromosome inactivation (XCI) is an important mechanism that compensates for the difference in gene dosage between XX females and XY males in mammals. Interestingly, the XCI in humans appears to start from the 8-cell stage [23]. Moreover, embryonic left-right separation is an important event during embryogenesis of bilaterians. In a recent study, by analyzing multiple lines of molecular and cell biology evidence, Ma concluded that embryos of bilaterians are divided into left and right lateral halves at or shortly after 8-cell stage [24].

*AZIN1* plays a role in cell growth and proliferation by maintaining polyamine homeostasis within cells [25]. A non-synonymous editing (A1099G) on *AZIN1* was found to be conserved in human and mouse [11]. Previous study showed that the A1099G edited *AZIN1* resulted in substantially enhancing cell proliferation in cultured liver cell lines [25]. In addition, previous studies showed that the editing frequencies at this site were significantly higher in tumors than in matched nontumorous tissues in hepatocellular carcinoma and esophageal squamous cell carcinoma [25, 26]. Besides, *AZIN1* expression was significantly increased in esophageal tumors compared with their matched nontumor specimens [26]. We observed that A1099G edited *AZIN1* are present in 31 out of 38 cells crossing from oocyte to 4-cell stages, and 4 out of 20 cells at 8-cell stages. Intriguingly, the editing frequency at this site dramatically dropped from 4-cell to 8-cell stage while the *AZIN1* expression substantially increased. We found that *AZIN1* expression was highly and negatively correlated with the A1099G editing frequencies. As either increase in A1099G editing or high expression of *AZIN1* may promote cell proliferation, we speculate that the frequent A1099G editing in earlier stages may be a compensation of the low level of *AZIN1* expression. When embryos produce more *AZIN1*, a decrease in edited *AZIN1* could keep the stability of overall *AZIN1* activity. We believe that the underlying biological significance of the frequent A1099G editing on *AZIN1* and the function of the A1099G edited *AZIN1* during human early embryogenesis requires further investigation. We also believe that the negative association between the *AZIN1* expression and the A1099G editing frequencies during early human embryogenesis is particularly interesting, deserving more comprehensive investigation.

## Conclusions

Taken together, our study indicates that human embryos undergo dynamic changes in genome-wide A-to-I RNA-editing during human early embryogenesis. Our findings underscore the importance of A-to-I RNA-editing during early human embryogenesis. It is worth noting that our findings are based on the observation of 68 cells from 29 embryos across 7 embryonic stages. Therefore we believe it is necessary to conduct more comprehensive studies to verify our findings and to understand the biological significance of the dynamic changes in A-to-I editing during human early embryogenesis.

## Methods

### Reads mapping and pre-processing

We downloaded single cell RNA-seq data of human embryos spanning from oocyte to morula stages from two previous studies [8, 9] from NCBI database. In both studies, RNA libraries were sequenced on Illumina platform but not strand-specific. Xue et al., sequenced the libraries as 90 bp-long pair-end reads, while Yan et al., obtained 100 bp-long single-end reads. We noticed that the sequencing quality near the 3’-ends of some single-end reads were not satisfactory. Therefore, we trimmed off 15 bp from the 3’-end of every single-end sequencing read to eliminate sequencing errors. We used SOAPnuke to filter out reads from both studies that contained adapters and low quality using default parameter. We downloaded the hg19 (GRCh37) genome sequences from the UCSC Genome Browser (http://genome.ucsc.edu). We aligned the filtered reads with Tophat2 [27] using command:

- Tophat2 --read-gap-length 3 --read-edit-dist 3 --no-novel-juncs --no-novel-indels --transcriptome-index = transcriptome-index hg19.
- The transcriptomes-index is generated using ensemble gene set. The command is:
- Tophat2 -G Homo_sapiens.GRCh37.75.gtf --transcriptome-index = transcriptome-index hg19.
- Then we removed PCR-induced duplications using Picard (v1.84; http://broadinstitute.github.io/picard/) and recalibrated base quality using GATK (v2.8-1).

### RNA-editing detection

We only selected the data of 68 single cells from 29 embryos each with over 0.5 Gb uniquely mapped bases for further analyses. We summarized the base calls of pre-processed aligned RNA-reads to the human reference in pileup format. To identify candidate RNA-editing sites, we only used sequencing bases with base-quality ≥ 20. We determined RNA-editing sites as follows:

1. We perform statistical tests based on binomial distribution B (n, p) to distinguish true variants from sequencing errors on every mismatch site, where p denotes the background mismatch rate of each transcriptome, and n denotes sequencing depth on this site. On a given specific site with k reads supporting variant in all n mapped reads, we use B (k, n, p) to calculate the probability that the k mismatches are all due to sequencing errors. This probability is adjusted using the Benjamini-Hochberg method. We only retain candidate sites with adjusted *P*-value < 0.01. In addition, the candidate variant sites should be with mapped reads ≥ 4, variant-supporting reads ≥3, and mismatch frequencies (variant-supporting-reads/mapped-reads) ≥ 0.1.
2. We filter out variants with strand bias, referring to abnormal distribution of sense strand variant supporting reads and antisense strand variant supporting reads in sequencing data. This sequencing bias may introduce false positive. We estimated strand bias and filtered out variants with strand bias as follows. (a) We performed a two-tailed Fisher’s exact test (FET) using the following two-by-two table:

**Table:** 

Sense strand variant supporting reads	Sense strand reference supporting reads
Antisense strand variant supporting reads	Antisense strand reference supporting reads
  (b) We estimated variant strand frequency (sense-strand variant-supporting reads divided by total variant-supporting reads), variant strand preference [absolute (variant strand frequency minus 0.5)], reference strand frequency (sense-strand reference minus supporting read number divided by total reference-supporting read number), and reference strand preference [absolute (reference strand frequency minus 0.5)]. (c) We filtered out variant sites displaying significant strand bias, defined as either FET *P*-value < 0.005 plus variant strand preference > reference strand preference, or variant strand frequency > 0.9, or variant strand frequency < 0.1.
3. Because the mismatches at ends of sequencing reads are less credible than the ones in the middle of reads, therefore, we filter out variants with position bias, i.e. the majority of variant supporting bases are located at read ends. Read end is defined as 10 bp at 3’-end or 5 bp at 5’-end. Position bias are defined as either FET *P*-value < 0.05 plus read end frequency > read middle frequency, or read end frequency > 0.9.
4. To reduce false positives introduced by misalignment of sequencing reads to high similarity regions of the reference sequences, we perform a realignment filtering. Specifically, we extract variant-supporting reads on candidate variant sites and realign them against a combination reference (hg19 genome + ensemble transcript reference v75) by bwa-0.6.2. We retain a candidate variant site if at least 90 % of its variant-supporting reads are realigned to this site.
5. We remove variant sites that are either in simple repeat regions (http://hgdownload.cse.ucsc.edu/goldenpath/hg19/database/), or in homopolymer regions (runs of ≥ 5 bp). For sites located in non-Alu regions, we additionally remove the sites within 5 bp from a splicing site.
6. To filter out common DNA SNPs, we build combined DNA SNP datasets from dbSNP (V138), 1000 Genome SNP (Phase 3), and human populations of Dutch [28], Mongolian (unpublished), and Dai (unpublished). Candidate variants are filtered out if they are found in the combined DNA SNP datasets.
7. For rare SNPs filtering, we filter out RNA variants that are only detected in one embryo, because true editing sites are often present in different individuals, whereas rare SNPs are most likely not, as Ramaswami suggested [10].
8. We also filter out candidate RNA variants if their mismatch frequencies > 0.95.

Finally, we tried to filter out private DNA-induced variants using the exon DNA sequencing data from the father of 12 embryos in Xue’s study. We found no additional paternal exonic DNA variants in the RNA mismatches we identified (Additional file 1: Table S6), suggesting that the false positive rate of DNA-induced RNA variants within the RNA mismatches we identified is considerably low.

### Hierarchical clustering

In cluster analysis, we created a matrix C (i, k) to store the editing frequency (0 for no edit and −1 for depth < 4) on site i in sample k. We calculated the euclidean distances among cells based on this matrix using the dist() function and performed hierarchical clustering using the hclust() function with default parameters. Both functions could be found in R package stats. The heatmap was drawn using R package pheatmap.

### Gene expression analyses

We calculate the number of reads aligned to each gene using the featureCounts function in R package Rsubread [29]. We performed differential gene expression analyses using the R package edgeR [30]. The RPKM values and differential expression *P*-values of genes used in this study were reported by edgeR.

### RNA-editing sites annotation

We used ANNOVAR [31, 32] to functionally annotate RNA-editing sites. The gene set used in annotation including ensemble gene set v75 and NONCODE v4 [33]. When studying the editing in miRNA-target regions, we focused on regions that were complementary to miRNA seed within 3’ untranslated regions based on the predicted miRNA targeted regions downloaded from http://www.microrna.org/microrna/getDownloads.do (August 2010 release, good mirSVR scores).

### Gene set enrichment analysis

The gene set enrichment analysis is performed on website http://software.broadinstitute.org/gsea/msigdb/annotate.jsp [34, 35]. The gene sets we used to compute overlaps included Canonical pathways (CP), BioCarta gene sets (CP:BIOCARTA), KEGG gene sets (CP:KEGG) and Reactome gene sets (CP:REACTOME).

## Additional files

Additional file 1: Figure S1. RNA sequencing data summary. **Figure S2.** Changes in RNA-editing levels during early human embryogenesis in each study. **Figure S3.** Changes in *ADAR* expression levels during early human embryogenesis. **Figure S4.** Changes in exonic editing frequency are negatively associated with the gene expression of *DCAF16, RPL23AP53* and *SNHG16*. **Figure S5.** Changes in editing sites in miRNA-targeted mRNA regions. **Table S2.** *ADAR* and *ADARB1* expression in different tissues revealed by Illumina Body Map project. **Table S6.** exonic DNA filter efficiency of the 17 cells from 12 embryos in Xue’s study. (DOC 775 kb) Additional file 2: Table S1. Candidate A-to-I RNA-editing sites. **Table S3.** Editing levels of A-to-I RNA-editing events that may recode the proteins. **Table S4.** Editing levels of A-to-I RNA-editing events in miRNA-targeted mRNA regions. **Table S5.** Significant GSEA results of the genes that are preferably edited in miRNA-targeted regions in cells crossing oocyte to 4-cell stages. (XLSX 582 kb)

## Abbreviations

GSEA: Gene set enrichment analysis
lincRNAs: Long intergenic noncoding RNAs
ncRNA: Noncoding RNA
RPKM: Reads Per Kilobase per Million mapped reads
XCI: X chromosome inactivation

## References

[1] Farajollahi S, Maas S (2010). Molecular diversity through RNA editing: a balancing act. Trends Genet.

[2] Avesson L, Barry G (2014). The emerging role of RNA and DNA editing in cancer. Biochim Biophys Acta.

[3] Savva YA, Rieder LE, Reenan RA (2012). The ADAR protein family. Genome Biol.

[4] Wang Q, Khillan J, Gadue P, Nishikura K (2000). Requirement of the RNA editing deaminase ADAR1 gene for embryonic erythropoiesis. Science.

[5] Liddicoat BJ, Piskol R, Chalk AM, Ramaswami G, Higuchi M, Hartner JC, Li JB, Seeburg PH, Walkley CR, Liddicoat BJ, Piskol R, Chalk AM, Ramaswami G, Higuchi M, Hartner JC, Li JB, Seeburg PH, Walkley CR (2015). RNA editing by ADAR1 prevents MDA5 sensing of endogenous dsRNA as nonself. Science.

[6] Shtrichman R, Germanguz I, Mandel R, Ziskind A, Nahor I, Safran M, Osenberg S, Sherf O, Rechavi G, Itskovitz-Eldor J (2012). Altered A-to-I RNA editing in human embryogenesis. PLoS One.

[7] Dobson AT, Raja R, Abeyta MJ, Taylor T, Shen S, Haqq C, Pera RAR (2004). The unique transcriptome through day 3 of human preimplantation development. Hum Mol Genet.

[8] Xue Z, Huang K, Cai C, Cai L, Jiang CY, Feng Y, Liu Z, Zeng Q, Cheng L, Sun YE (2013). Genetic programs in human and mouse early embryos revealed by single-cell RNA sequencing. Nature.

[9] Yan L, Yang M, Guo H, Yang L, Wu J, Li R, Liu P, Lian Y, Zheng X, Yan J (2013). Single-cell RNA-Seq profiling of human preimplantation embryos and embryonic stem cells. Nat Struct Mol Biol.

[10] Ramaswami G, Zhang R, Piskol R, Keegan LP, Deng P, O’Connell MA, Li JB (2013). Identifying RNA editing sites using RNA sequencing data alone. Nat Methods.

[11] Pinto Y, Cohen HY, Levanon EY (2014). Mammalian conserved ADAR targets comprise only a small fragment of the human editosome. Genome Biol.

[12] Fumagalli D, Gacquer D, Rothe F, Lefort A, Libert F, Brown D, Kheddoumi N, Shlien A, Konopka T, Salgado R (2015). Principles governing A-to-I RNA editing in the breast cancer transcriptome. Cell Rep.

[13] Bazak L, Levanon EY, Eisenberg E (2014). Genome-wide analysis of Alu editability. Nucleic Acids Res.

[14] Ramaswami G, Li JB (2014). RADAR: a rigorously annotated database of A-to-I RNA editing. Nucleic Acids Res.

[15] Petryszak R, Keays M, Tang YA, Fonseca NA, Barrera E, Burdett T, Fullgrabe A, Fuentes AM, Jupp S, Koskinen S (2016). Expression Atlas update--an integrated database of gene and protein expression in humans, animals and plants. Nucleic Acids Res.

[16] Han L, Diao L, Yu S, Xu X, Li J, Zhang R, Yang Y, Werner HM, Eterovic AK, Yuan Y (2015). The genomic landscape and clinical relevance of A-to-I RNA editing in human cancers. Cancer Cell.

[17] Lee WS, Wells RG, Sabbag RV, Mohandas TK, Hediger MA (1993). Cloning and chromosomal localization of a human kidney cDNA involved in cystine, dibasic, and neutral amino acid transport. J Clin Invest.

[18] Evers R, Grummt I (1995). Molecular coevolution of mammalian ribosomal gene terminator sequences and the transcription termination factor TTF-I. Proc Natl Acad Sci.

[19] Potts PR, Yu H (2005). Human MMS21/NSE2 is a SUMO ligase required for DNA repair. Mol Cell Biol.

[20] Zhu Y, Yu M, Li Z, Kong C, Bi J, Li J, Gao Z, Li Z. ncRAN, a newly identified long noncoding RNA, enhances human bladder tumor growth, invasion, and survival. Urology. 2011;77(2):510. e511-510. e515. 10.1016/j.urology.2010.09.022 21147498

[21] Borchert GM, Gilmore BL, Spengler RM, Xing Y, Lanier W, Bhattacharya D, Davidson BL (2009). Adenosine deamination in human transcripts generates novel microRNA binding sites. Hum Mol Genet.

[22] Mo F, Wyatt AW, Sun Y, Brahmbhatt S, McConeghy BJ, Wu C, Wang Y, Gleave ME, Volik SV, Collins CC (2014). Systematic identification and characterization of RNA editing in prostate tumors. PLoS One.

[23] van den Berg IM, Laven JSE, Stevens M, Jonkers I, Galjaard R-J, Gribnau J, Hikke van Doorninck J (2009). X chromosome inactivation is initiated in human preimplantation embryos. Am J Hum Genet.

[24] Ma K (2013). Embryonic left-right separation mechanism allows confinement of mutation-induced phenotypes to one lateral body half of bilaterians. Am J Med Genet A.

[25] Chen L, Li Y, Lin CH, Chan TH, Chow RK, Song Y, Liu M, Yuan YF, Fu L, Kong KL (2013). Recoding RNA editing of AZIN1 predisposes to hepatocellular carcinoma. Nat Med.

[26] Qin Y-R, Qiao J-J, Chan THM, Zhu Y-H, Li F-F, Liu H, Fei J, Li Y, Guan X-Y, Chen L, Qin Y-R, Qiao J-J, Chan THM, Zhu Y-H, Li F-F, Liu H, Fei J, Li Y, Guan X-Y, Chen L (2014). Adenosine-to-Inosine RNA editing mediated by ADARs in esophageal squamous cell carcinoma. Cancer Res.

[27] Kim D, Pertea G, Trapnell C, Pimentel H, Kelley R, Salzberg SL (2013). TopHat2: accurate alignment of transcriptomes in the presence of insertions, deletions and gene fusions. Genome Biol.

[28] Liao Y, Smyth GK, Shi W (2014). Feature Counts: an efficient general purpose program for assigning sequence reads to genomic features. Bioinformatics.

[29] Robinson MD, McCarthy DJ, Smyth GK (2010). edgeR: a Bioconductor package for differential expression analysis of digital gene expression data. Bioinformatics.

[30] Xie C, Yuan J, Li H, Li M, Zhao G, Bu D, Zhu W, Wu W, Chen R, Zhao Y (2014). NONCODEv4: exploring the world of long non-coding RNA genes. Nucleic Acids Res.

[31] Francioli LC, Androniki M, Pulit SL, van Dijk F, Pier Francesco P, Elbers CC, Neerincx PBT, Kai Y, Victor G, Kloosterman WP, Patrick D, Abdel A, van Leeuwen EM, van Oven M, Martijn V, Mingkun L, Laros JFJ, Karssen LC, Alexandros K, Najaf A, Jouke Jan H, Eric-Wubbo L, Mathijs K, Martijn D, Heorhiy B, van Setten J, van Schaik BDC, Jan B, Nijman IJ, Ivo R, Tobias M, Alexander S, Hehir-Kwa JY, Handsaker RE, Paz P, Mashaal S, Dana V, Fereydoun H, van Enckevort D, Hailiang M, Vyacheslav K, Moed MH, van der Velde KJ, Fernando R, Karol E, Carolina M-G, Aaron I, McCarroll SA, Marian B, de Craen AJM, Suchiman HED, Albert H, Ben O, Uitterlinden AG, Gonneke W, Mathieu P, Veldink JH, van den Berg LH, Pitts SJ, Shobha P, Purnima S, Cox DR, Sunyaev SR, den Dunnen JT, Mark S, de Knijff P, Manfred K, Qibin L, Yingrui L, Yuanping D, Ruoyan C, Hongzhi C, Ning L, Sujie C, Jun W, Bovenberg JA, Itsik P'e, Eline Slagboom P, van Duijn CM, Boomsma DI, van Ommen G-JB, de Bakker PIW, Swertz MA, Cisca W, LifeLines Cohort Study (2014). Whole-genome sequence variation, population structure and demographic history of the Dutch population. Nature Genetics.

[32] Wang K, Li M, Hakonarson H, Wang K, Li M, Hakonarson H (2010). ANNOVAR: functional annotation of genetic variants from high-throughput sequencing data. Nucleic Acids Research.

[33] Yang H, Wang K, Yang H, Wang K (2015). Genomic variant annotation and prioritization with ANNOVAR and wANNOVAR. Nature Protocols.

[34] Mootha VK, Lindgren CM, Eriksson K-F, Subramanian A, Sihag S, Lehar J, Puigserver P, Carlsson E, Ridderstråle M, Laurila E, Houstis N, Daly MJ, Patterson N, Mesirov JP, Golub TR, Tamayo P, Spiegelman B, Lander ES, Hirschhorn JN, Altshuler D, Groop LC. PGC-1Î±-responsive genes involved in oxidative phosphorylation are coordinately downregulated in human diabetes. Nature Genetics. 34(3):267–73 10.1038/ng1180 12808457

[35] Subramanian A, Tamayo P, Mootha VK, Mukherjee S, Ebert BL, Gillette MA, Paulovich A, Pomeroy SL, Golub TR, Lander ES, Mesirov JP, Subramanian A, Tamayo P, Mootha VK, Mukherjee S, Ebert BL, Gillette MA, Paulovich A, Pomeroy SL, Golub TR, Lander ES, Mesirov JP (2005). Gene set enrichment analysis: A knowledge-based approach for interpreting genome-wide expression profiles. Proceedings of the National Academy of Sciences.

