# Supplementary material for: Single-cell RNA sequencing reveals dynamic changes in A-to-I RNA editome during early human embryogenesis
Source: BMC Genomics. 2016 Sep 29;17:766. doi: 10.1186/s12864-016-3115-2 (PMC5043600; doi:10.1186/s12864-016-3115-2)
Supplement: Additional file 1: Figure S1. — RNA sequencing data summary. Figure S2. Changes in RNA-editing levels during early human embryogenesis in each study. Figure S3. Changes in ADAR expression levels during early human embryogenesis. Figure S4. Changes in exonic editing frequency are negatively associated with the gene expression of DCAF16, RPL23AP53 and SNHG16. Figure S5. Changes in editing sites in miRNA-targeted mRNA regions. Table S2. ADAR and ADARB1 expression in different tissues revealed by Illumina Body Map project. Table S6. exonic DNA filter efficiency of the 17 cells from 12 embryos in Xue’s study. (DOC 775 kb) [file 12864_2016_3115_MOESM1_ESM.doc]

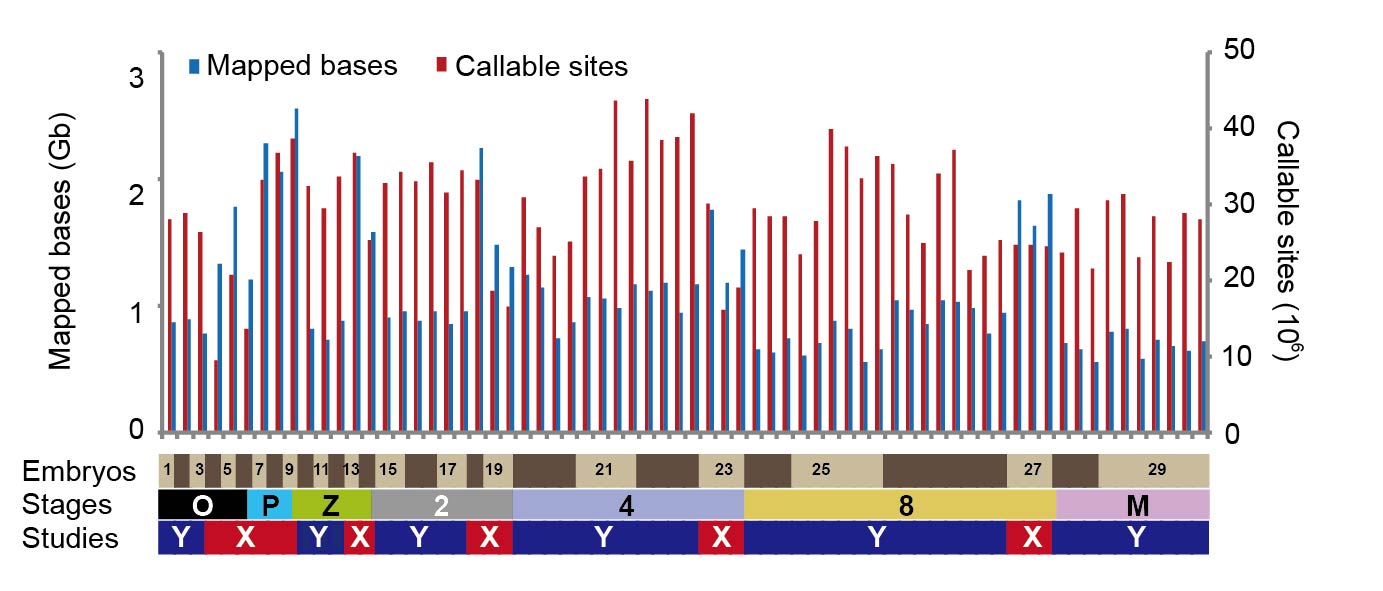


**Figure S1 RNA sequencing data summary.** The 68 cells isolated from 29 embryos were denoted by numbers. Each bar represents one cell. O: oocyte; P: pronucleus; Z: zygote; 2: 2-cell; 4: 4-cell; 8: 8-cell; M: morula. Y: data from Yan *et al.*; X: data from Xue *et al.*


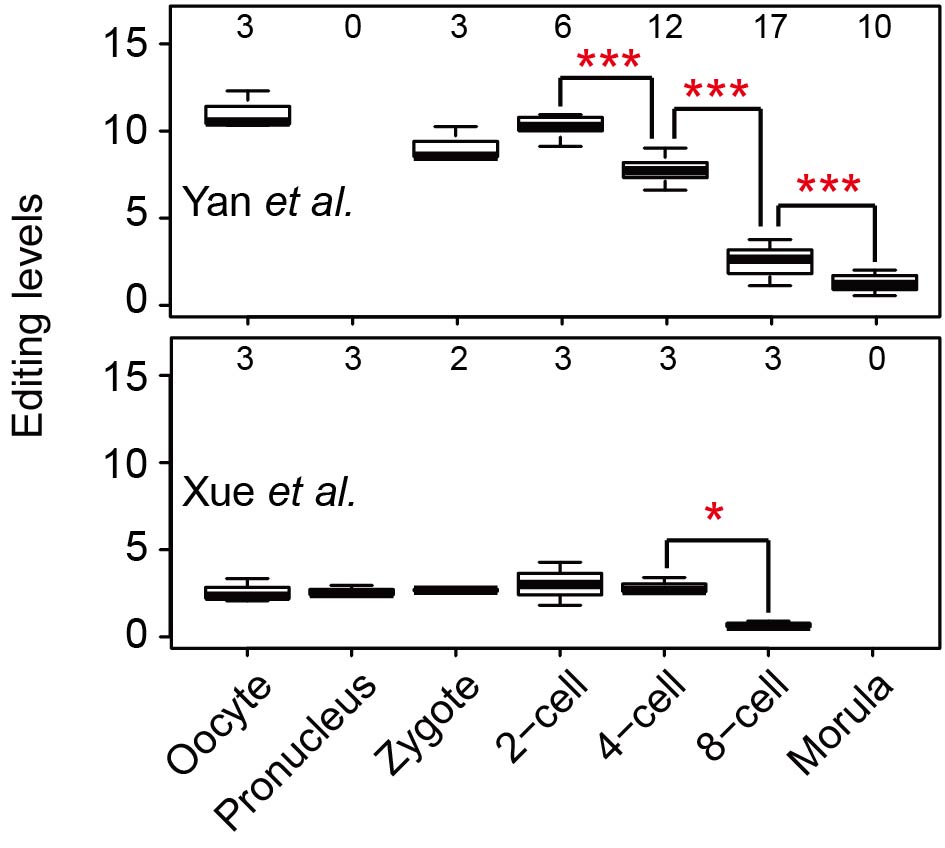


**Figure S2 Changes in RNA-editing levels during early human embryogenesis in each study.** * P < 0.05; ** P < 0.01; *** P < 0.001 (Wilcoxon rank sum test). The digit above each box represents the cell number of the stage in each study.


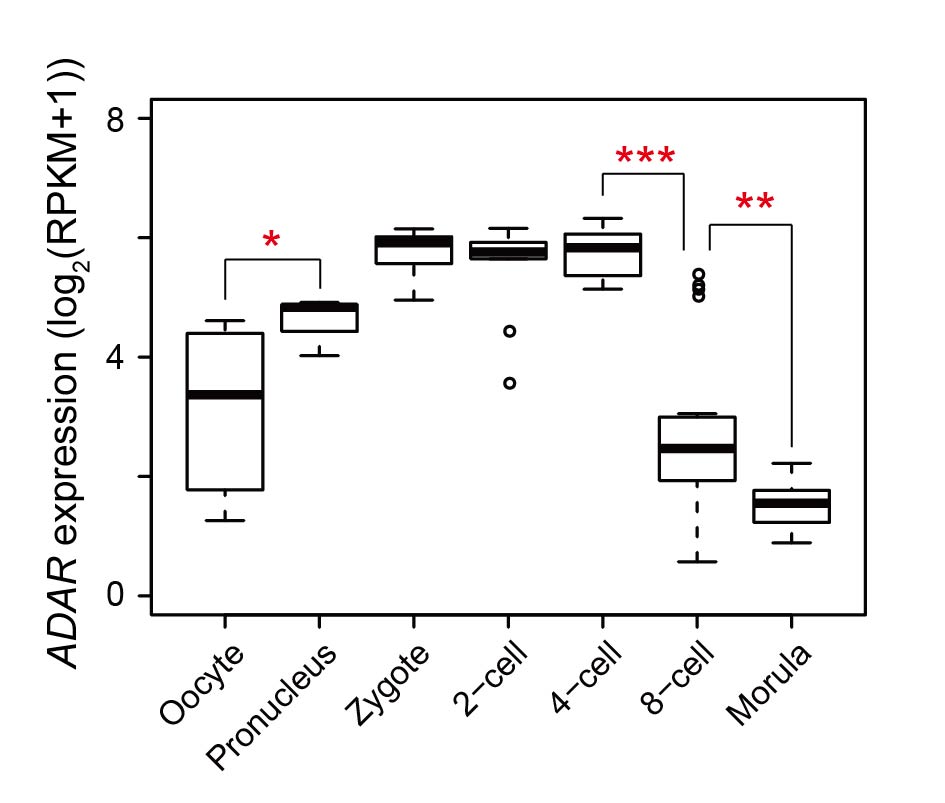


**Figure S3 Changes in *ADAR* expression levels during early human embryogenesis.** * *P* < 0.05; ** *P* < 0.01; *** *P* < 0.001 (edgeR)

**
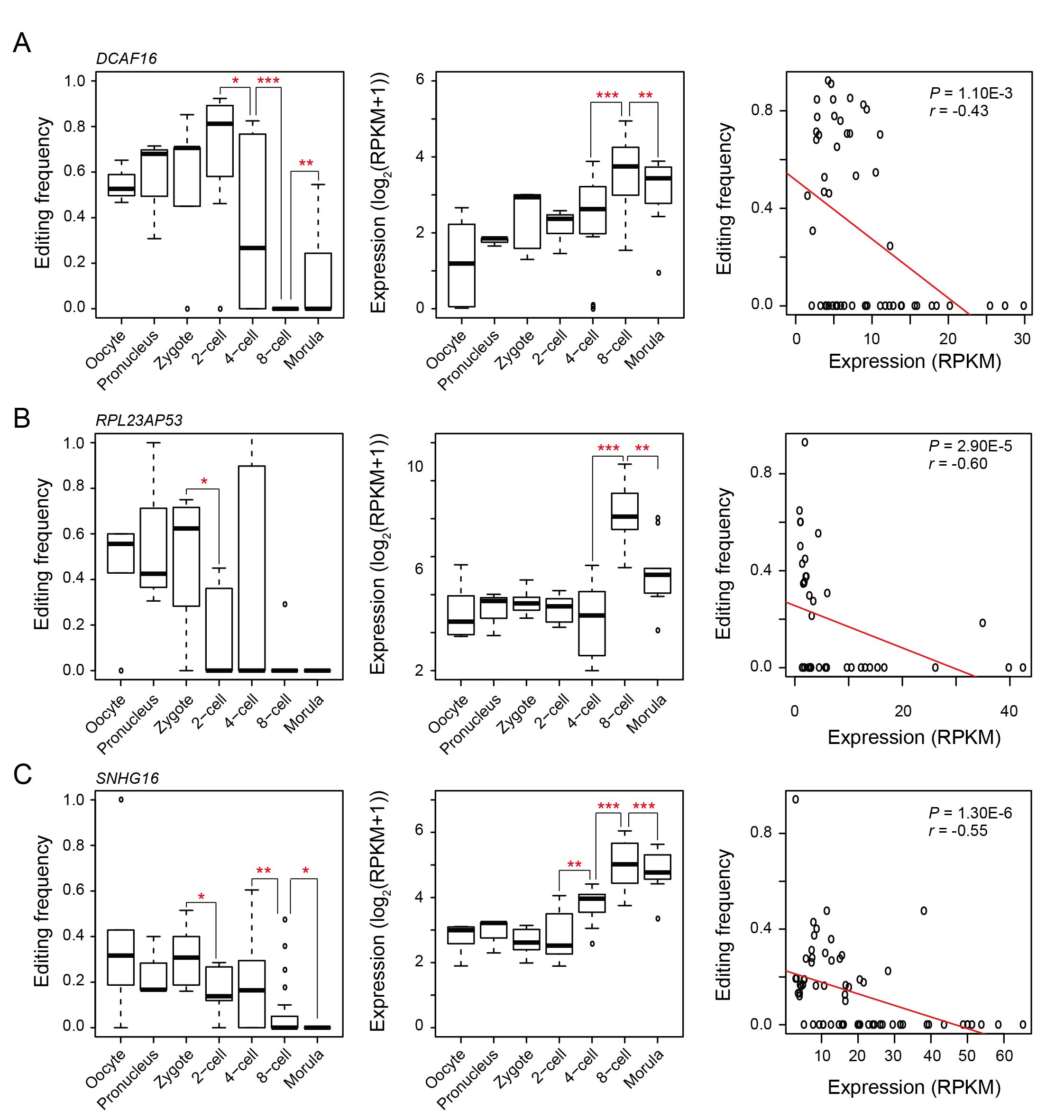
**

**Figure S4 Changes in exonic editing frequency are negatively associated with the gene expression of *SRP14*, *DCAF16* and *SNHG16*.** Changes in editing frequency (left) and gene expression (middle) of *SRP14* **(A)**, *DCAF16* **(B)**, and *SNHG16* **(C)** during early human embryogenesis. Averaged editing frequency is negatively associated with the gene expression (right) of *SRP14* **(A)**, *DCAF16* **(B)**, and *SNHG16* **(C)**, respectively. Each open circle represents one embryonic stage in figures on the right. For *SNHG16*, the editing frequency in each cell is the average editing frequency of all the exonic editing in *SNHG16* in the cell.* *P* < 0.05; ** *P* < 0.01; *** *P* < 0.001. SNHG16


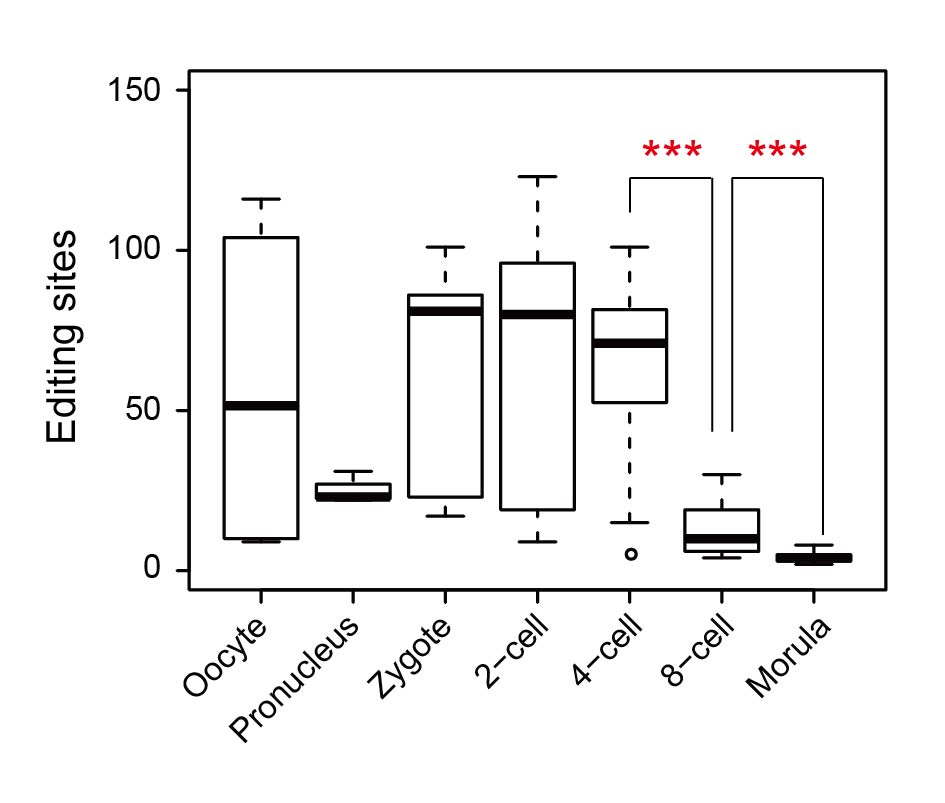


**Figure S5 Changes in editing sites in miRNA-targeted mRNA regions.**

**Table S2. *ADAR* and *ADARB1*expression in different tissues revealed by Illumina Body Map project.**

| **Tissues** | **Gene expression** | | **Fold Change** |
| --- | --- | --- | --- |
|  | ***ADAR*** | ***ADARB1*** |  |
| adipose | 31 | 4 | 8 |
| adrenal | 44 | 6 | 11 |
| brain | 31 | 5 | 8 |
| breast | 22 | 4 | 6 |
| colon | 20 | 8 | 5 |
| heart | 11 | 1 | 3 |
| kidney | 27 | 3 | 7 |
| leukocyte | 94 | 2 | 24 |
| liver | 19 | 1 | 5 |
| lung | 30 | 12 | 8 |
| lymph node | 49 | 9 | 12 |
| ovary | 56 | 4 | 14 |
| prostate | 30 | 19 | 8 |
| skeletal muscle | 6 | 2 | 2 |
| testis | 33 | 5 | 8 |
| thyroid | 28 | 2 | 7 |

**Table S6. exonic DNA filter efficiency of the 17 cells from 12 embryos in Xue’s study.**

| **Cells** | **Exonic DNA variants before filter** | **Exonic DNA variants after filter** |
| --- | --- | --- |
| SRR893046 | 458 | 0 |
| SRR893047 | 966 | 0 |
| SRR893048 | 528 | 0 |
| SRR893049 | 2,443 | 0 |
| SRR893050 | 2,392 | 0 |
| SRR893051 | 2,819 | 0 |
| SRR893052 | 2,041 | 0 |
| SRR893053 | 1,223 | 0 |
| SRR893054 | 2,374 | 0 |
| SRR893055 | 885 | 0 |
| SRR893056 | 742 | 0 |
| SRR893057 | 2,465 | 0 |
| SRR893058 | 852 | 0 |
| SRR893060 | 1,107 | 0 |
| SRR893061 | 1,730 | 0 |
| SRR893062 | 1,655 | 0 |
| SRR893063 | 1,696 | 0 |
